# Comparison of Corneal Higher-Order Aberrations Following Topography-Guided LASIK and SMILE for Myopic Correction: A Propensity Score Matching Analysis

**DOI:** 10.3390/jcm11206171

**Published:** 2022-10-19

**Authors:** Eun Min Kang, Ik Hee Ryu, In Sik Lee, Jin Kuk Kim, Sun Woong Kim, Yong Woo Ji

**Affiliations:** 1 B & VIIT Eye Center, Seoul 06615, Korea; 2 Department of Ophthalmology, Yonsei University Wonju College of Medicine, Wonju 26426, Korea; 3 Department of Ophthalmology, Yongin Severance Hospital, Yonsei University College of Medicine, Yongin 16995, Korea

**Keywords:** topography-guided laser-assisted in situ keratomileusis, small-incision lenticule extraction, higher-order aberration, Contoura LASIK, propensity score matching

## Abstract

Ocular aberrations, particularly corneal higher-order aberrations (HOAs), which impair visual quality, should be minimized or corrected during any laser vision correction. We compared changes in visual outcomes, including HOAs, in patients who underwent Topography-Guided laser-assisted in situ keratomileusis (TG-LASIK) or small-incision lenticule extraction (SMILE) after propensity score matching (PSM) to reduce selection bias. Of 2749 patients who underwent SMILE or TG-LASIK for myopia, 152 eyes underwent complete ophthalmic examination preoperatively and over six months postoperatively. Visual outcomes were comparatively analyzed after PSM. As a result, 45 eyes were included in each group after PSM. There was a comparable improvement in visual acuity (VA) and refractive parameters postoperatively, with no difference between the two PSM-groups. However, 6.6% in the SMILE PSM-group lost two or more lines of Snellen VA at the six-month follow-up, while none in the TG-LASIK PSM-group did. Specifically, the SMILE PSM-group showed a significant increase in corneal HOAs, including spherical aberration, coma, and total HOAs (0.0736 ± 0.162 μm; 0.181 ± 0.233 μm; and 0.151 ± 0.178 μm, respectively), whereas TG-LASIK PSM-group did not. Furthermore, SMILE PSM-group had greater postoperative corneal HOAs than those in TG-LASIK PSM-group. Collectively, TG-LASIK induces fewer corneal HOAs even after facilitating between-group comparability using PSM analysis. TG-LASIK provides better visual quality than SMILE for myopia.

## 1. Introduction

Ocular aberrations, including various higher-order aberrations (HOAs), compromise retinal image quality [1]. Alterations in corneal shape (from prolate to oblate) following surgery increase some HOAs, such as spherical aberration (SA) and coma [2,3,4,5]. HOAs cause night vision glare, photophobia, blurred vision, and decreased contrast sensitivity, and consequently compromise postoperative visual acuity [1,5]. In particular, corneal HOAs play a critical role in determining total postoperative aberrations even though induced anterior aberrations can be partly compensated by internal aberrations [6,7]. 

Currently, it is possible to correct not only lower-order aberrations, but also HOAs using a customized ablation pattern [1,2,6,8,9]. Topography-guided LASIK (TG-LASIK) with primary refractive correction (WaveLight^®^ Contoura; WaveLight Laser Technologie AG, Erlangen, Germany) has been introduced through US Food and Drug Administration (FDA) approval [10,11]. TG-LASIK attempts to create a more uniform corneal shape to remove HOAs during refractive error correction [12,13,14]. Reportedly, TG-LASIK induces fewer HOAs and decreases ocular trefoil, coma, and corneal total HOAs [2,15,16].

Small-incision lenticule extraction (SMILE) is a flapless refractive surgical method of intrastromal keratomileusis that removes the corneal stromal lenticule through a small corneal incision [8,17,18]. SMILE has achieved comparable efficacy, safety, and predictability for myopic correction [19,20,21,22,23,24]. Previous studies have also demonstrated that SMILE induces fewer total HOAs compared with other conventional laser surgeries including femtosecond laser-assisted LASIK (FS-LASIK) [3,4,25]. Otherwise, SMILE is thought to result in inferior or not superior visual outcomes compared with TG-LASIK even though surgical indications and surgeon preferences are different [8,14]. However, there are few studies comparing HOAs between these techniques.

The present study aimed to compare changes in visual outcomes, particularly corneal HOAs, after TG-LASIK and SMILE for myopic correction. To eliminate confounding factors from patient’s characteristics or physician’s treatment preference, we used propensity score matching (PSM) analysis when estimating the effect of TG-LASIK and SMILE on treatment outcomes.

## 2. Materials and Methods

### 2.1. Patients

Patients who underwent either TG-LASIK or SMILE at the B & VIIT Eye Center (Seoul, Korea) between January and August 2018 were included in this study. Inclusion criteria consisted of a minimum age of 19 years, stable refraction for at least one year, corrected distance visual acuity (CDVA) of 20/25 or better (0.1 logMAR or less), no other ocular conditions except myopia of at least 7.0 diopters and astigmatism ranging from 0 to 3.5 diopters, and a minimum corneal thickness of 480 µm. Patients lacking a six-month postoperative follow-up were excluded. This study was approved by the institutional review board and conducted in accordance with the tenets of the Declaration of Helsinki. Informed consent was obtained from all patients.

### 2.2. Preoperative Examination

A complete ophthalmic examination was performed to screen for corneal abnormalities and determine patient eligibility for refractive surgery. Preoperative examination included objective and manifest visual refraction, CDVA, intraocular pressure (IOP) and noncontact pachymetry (TONOPACHY™ NT-530P; NIDEK, Gamagori, Japan), pupil size (NIDEK Pupillometer), and slit-lamp and fundus examination. Regular topographic patterns of total cornea were confirmed with the Pentacam HR (OCULUS Optikgerate GmbH, Wetzlar, Germany). All clinical refractions were obtained at a vertex distance of 12.0 mm and were performed by trained optometrists. Patients discontinued wearing contact lenses for at least seven days (soft lenses) or two weeks (hard lenses) before the assessment.

### 2.3. Surgical Technique 

#### 2.3.1. TG-LASIK Procedure

All surgical procedures were performed by experienced surgeons. Four to eight corneal topographies were acquired on un-dilated eyes with the WaveLight Topolyzer VARIO (Alcon Laboratories, Fort Worth, TX, USA). Images were reviewed for quality. Criteria for image acceptance were as follows: appropriate recognition of the mire edge and pupil by the software, the absence of significant missing data, minimal disruptions of mires, and a low median absolute deviation variability score below 0.10. Images that did not meet the criteria were excluded. The remaining images were exported to the Contoura software (WaveLight Laser Technologie AG, Erlangen, Germany) to create ablation profiles. The planned ablation profiles were then examined with the sphere and cylinder set to zero, showing only the anterior corneal HOA ablation profile. The HOA pattern was confirmed to match the anterior elevation topography without artifacts affecting the ablation pattern. The clinically measured manifest refraction sphere and refractive astigmatism magnitude were entered into the Contoura software as treatment parameters. A custom nomogram developed using a large electronic medical record database was used to modify the sphere and cylinder. The corneal flap was created with the VisuMax FS laser system (Carl Zeiss Meditec AG, Jena, Germany) using a pulse of 140 nJ and a repetition rate of 500 kHz. The position and angle of the hinge were set at the 12 o’clock position and at 90°, respectively. The intended diameter and flap thickness were 8 mm and 100 µm, respectively. The track and spot distances were set as 4.0 µm for flap creation and 2 mm for the flap side cut. After lifting the flap, the WaveLight EX500 excimer laser with Contoura software was used to perform stromal ablation (6.5 mm zone), after which the flap was carefully repositioned over the stroma.

#### 2.3.2. SMILE Procedure

SMILE was performed using a VisuMax FS laser. A custom nomogram developed using a large electronic medical record database was used to modify the sphere and cylinder. FS laser pulses (500 kHz, laser energy range of 115 to 135 nJ) were focused in a spiral pattern with a spot distance of 4.0 µm to cut the tissue. The diameter of the cap was 7.5 mm, and the optical zone diameter was between 6.2 and 6.7 mm. The intended cap thickness was 110–120 µm. A 2 mm incision was made at the 145° meridian. The upper interface and lower layer were dissected. Once both layers had been separated, the lenticule was removed from the cornea. 

At the end of the two procedures, the cornea was irrigated with saline, and all patients received one drop of moxifloxacin. The postoperative regimen included loteprednol and moxifloxacin eye drops for two weeks. 

### 2.4. Postoperative Examination 

A postoperative follow-up was conducted the day after surgery, and complete ophthalmic examination was conducted at six months postoperatively. The examination included uncorrected distance visual acuity (UDVA), manifest refractions, noncontact tonometry, central corneal thickness, keratometry, a slit-lamp examination, and Pentacam tomography. All postoperative complications were noted.

### 2.5. Measurement of HOAs

Corneal HOAs were measured at the 6.0 mm zone under standard scotopic light settings using the Pentacam (OCULUS). Root mean square values for SA (Z^0^_4_), coma (Z^1^_3_, Z^−1^_3_), and residual HOAs (from third order up) measured over the total cornea were used. The Pentacam scans were performed preoperatively and at the 6-month follow-up. All measurements were obtained by an experienced operator. Scans not meeting acceptable criteria (blinks during the scan or other artifacts) according to the Pentacam software indications, were repeated. Only scans graded as “OK” by the instruments were used for further analysis.

### 2.6. Statistical Analysis 

Propensity score matching analysis using R software (ver. 3.3.3; R Development Core Team, Vienna, Austria) and SAS (ver. 9.4; SAS Institute, Cary, NC, USA) was used to reduce potential selection bias associated with the retrospective observational study design. The propensity score was computed using a multivariate logistic regression model that included age, sex, manifest refraction and preoperative HOAs. We used the 1:1 nearest-neighbor technique with a caliper of 1.0 on the probability scale; replacement of the control was not permitted. 

Categorical variables are expressed as the frequency (percentage) and continuous variables are expressed as the mean and standard deviation. A paired *t*-test was performed to determine statistically significant differences between the two groups after PSM. The chi-square test was used for qualitative data and for comparing percentages. A paired *t*-test was used to compare pre- and postoperative HOAs and visual acuity changes for each PSM-group. A *p*-value of < 0.05 was considered to be significant.

## 3. Results

### 3.1. Preoperative Characteristics of Both Surgical Groups Both Surgical Procedures after Propensity Score Matching

**Figure 1:**
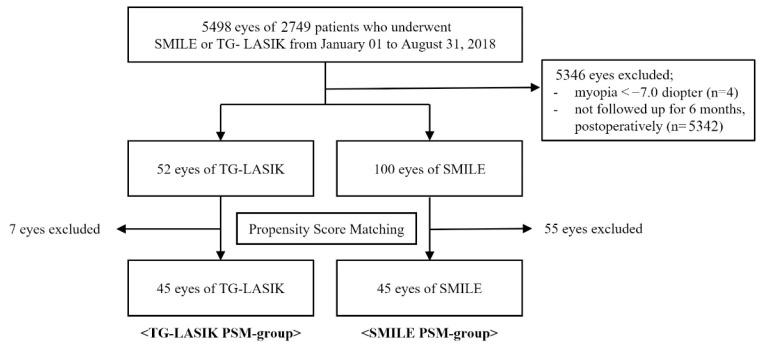
Flowchart showing patients receiving TG-LASIK and SMILE for propensity score matching analysis (TG-LASIK, topography-guided laser-assisted in situ keratomileusis; SMILE, small-incision lenticule extraction; PSM, propensity score matching).

Medical records for a total of 5498 eyes of 2749 patients who underwent SMILE or TG-LASIK during the study period were retrieved. Of them, 5346 eyes were excluded due to myopia greater than −7.00 diopters (four eyes) and loss of postoperative follow-up (5342 eyes). Following the PSM analysis of the remaining 152 eyes, 45 eyes in both the TG-LASIK and SMILE groups each, were subjected to further analysis (Figure 1). There were no complications during the six-month postoperative period.

Before PSM, there was no significant difference in age, sex, or spherical equivalent (SEQ) between the two treatment groups. However, the TG-LASIK group showed significantly more astigmatism and total HOAs compared with the SMILE group (−2.18 ± 1.02 vs. −0.74 ± 0.61 diopters; *p* < 0.001; and 0.65 ± 0.59 vs. 0.45 ± 0.13 μm, respectively; *p* < 0.05). After PSM, there was no significant difference in any preoperative characteristic (Table 1).

### 3.2. Comparison of Refractive Outcome and Visual Acuity between Two Propensity-Score-Matched Groups after Each Surgical Procedures

**Figure 2:**
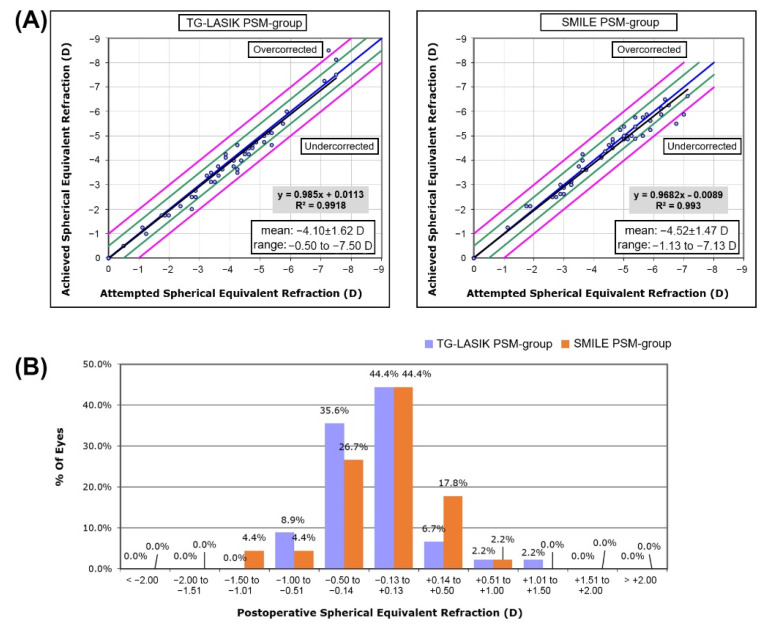
(**A**) The attempted versus achieved SEQ scatterplot in each TG-LASIK and SMILE propensity-score-matched group. (**B**) Changes in postoperative SEQ compared with preoperative CDVA in TG-LASIK and SMILE propensity-score-matched groups. (SEQ, spherical equivalent; TG-LASIK, topography-guided laser-assisted in situ keratomileusis; SMILE, small-incision lenticule extraction).

At six months after surgery, both PSM-groups showed definite improvement in visual acuity, spherical errors, astigmatism, and SEQ compared with their preoperative status (Table 2). The attempted versus achieved SEQ scatterplot revealed a high predictability in both TG-LASIK and SMILE PSM-groups with R^2^ values of 0.99 (Figure 2A). A similar percentage of TG-LASIK and SMILE eyes exhibited a SEQ within 0.50 and 1.00 D of the intended target (86.7% vs. 88.9% and 97.8% vs. 97.8%, respectively; Figure 2B).

**Figure 3:**
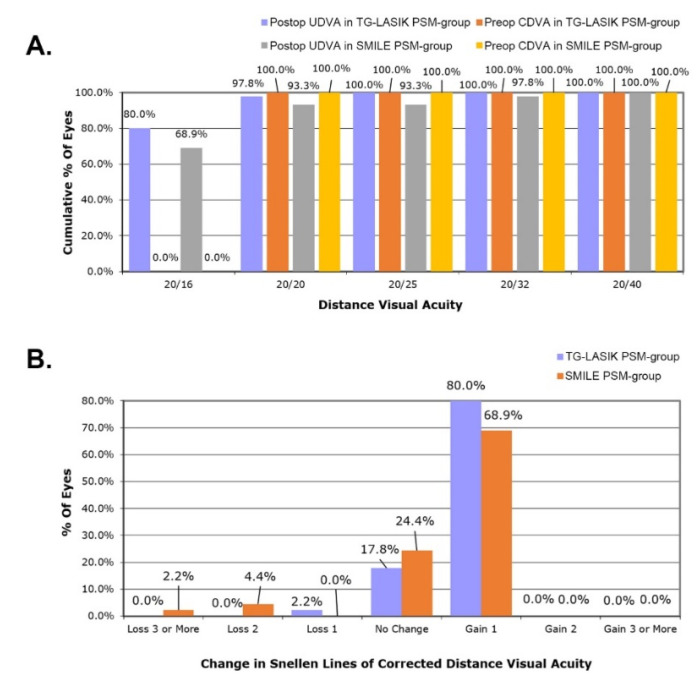
(**A**) Distance visual acuity following TG-LASIK and SMILE. (**B**) Changes in postoperative Snellen lines of CDVA compared with preoperative CDVA in TG-LASIK and SMILE propensity-score-matched groups. (TG-LASIK, topography-guided laser-assisted in situ keratomileusis; SMILE, small incision lenticule extraction; UDVA, uncorrected distance visual acuity; CDVA, corrected distance visual acuity).

There was no significant difference in the postoperative UDVA and SEQ between the TG-LASIK and SMILE PSM-groups, but there was a statistical tendency for TG-LASIK to be associated with better postoperative visual acuity (−0.0722 ± 0.0627 logMAR vs. −0.0367 ± 0.121 logMAR, respectively; *p* = 0.087; Table 2). Furthermore, a greater number of TG-LASIK eyes achieved a cumulative postoperative UDVA of 20/16 (80.0% vs. 68.9%), 20/20 (97.8% vs. 93.3%), 20/25 (100.0% vs. 93.3%), and 20/32 (100.0% vs. 97.8%) compared with SMILE eyes (Figure 3A). In the TG-LASIK PSM-group, 97.8% had no change or gained lines of CDVA, compared with 93.3% in the SMILE PSM-group (*p* < 0.05). In addition, compared with TG-LASIK eyes, 6.6% more SMILE eyes lost two or more lines of CDVA (Figure 3B).

### 3.3. Changes in Astigmatism and Corneal Aberration after Surgical Procedures

**Figure 4:**
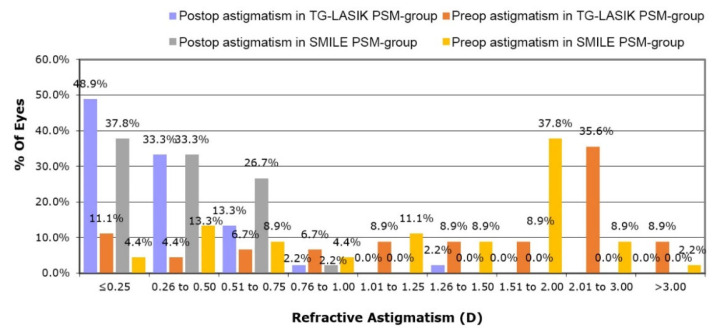
Changes in postoperative Snellen lines of CDVA compared with preoperative CDVA in TG-LASIK and SMILE propensity-score-matched groups. (TG-LASIK, topography-guided laser-assisted in situ keratomileusis; SMILE, small-incision lenticule extraction; UDVA, uncorrected distance visual acuity; CDVA, corrected distance visual acuity).

In terms of refractive astigmatism accuracy of each procedure, more eyes in the TG-LASIK PSM-group were within ±0.25 and ±0.50 diopters of the intended plano cylinder compared with those in the SMILE PSM-group (48.9% vs. 37.8% and 82.2% vs. 71.1%, respectively; *p* < 0.05; Figure 4), whereas the percentage of eyes within ±0.75 diopters of the intended plano cylinder were not significantly different between groups (95.6% vs. 97.8%, respectively; Figure 4). Although not significant, a greater number of TG-LASIK eyes had residual astigmatism of 0.75 D or greater, compared with SMILE eyes (4.4% vs. 2.2%, respectively; Figure 4).

**Figure 5:**
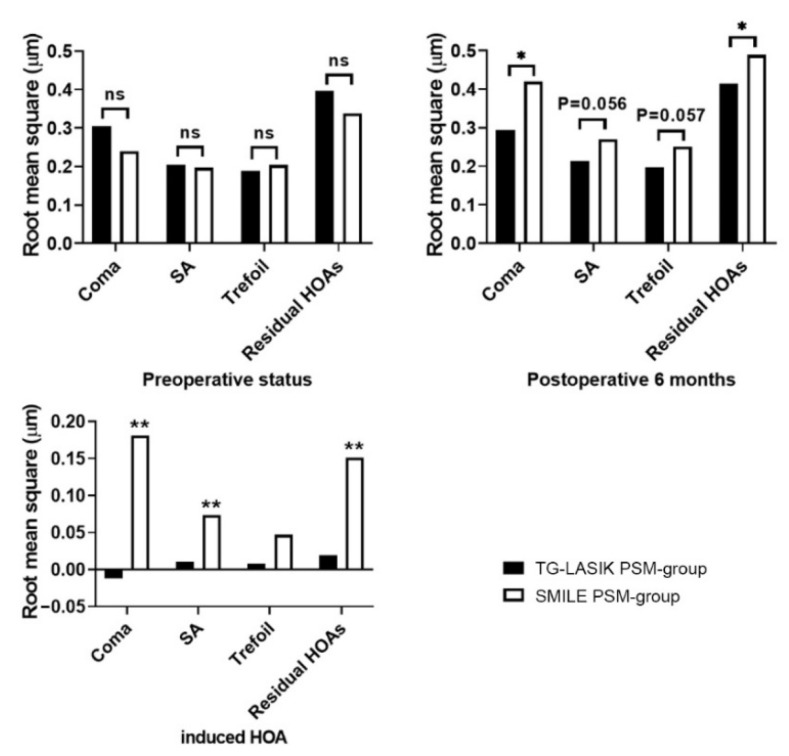
Comparison of corneal HOAs in TG-LASIK and SMILE propensity-score-matched groups. (TG-LASIK, topography-guided laser-assisted in situ keratomileusis; SMILE, small-incision lenticule extraction; SA, spherical aberration; HOA, higher-order aberration; * *p* < 0.05; ** *p* < 0.01; ns: not significant).

Figure 5 and Table 3 show the changes in corneal aberrations due to surgery. SMILE induced a significant increase in SA, coma, and residual HOAs (from third order up) (0.0736 ± 0.162 µm, *p* = 0.004; 0.181 ± 0.233 µm, *p* < 0.001; and 0.151 ± 0.178 µm, *p* < 0.001; respectively), whereas TG-LASIK induced no significant changes in HOAs after surgery. The coma of HOAs was even reduced after TG-LASIK; however, the changes were not statistically significant, and approached zero. Six months after surgery, there was no significant difference in SA between the TG-LASIK and SMILE PSM-groups, but SMILE eyes had a statistical tendency to exhibit more postoperative SA (*p* = 0.057). TG-LASIK eyes also showed significantly lower coma and residual HOAs compared with SMILE eyes. (*p* = 0.009 and *p* = 0.028, respectively).

## 4. Discussion

Studies comparing corneal HOAs induced by TG-LASIK and SMILE surgery have not been published. To the best of our knowledge, the present study is the first study to compare the changes in corneal HOAs after TG-LASIK and SMILE surgery matched for age, sex, preoperative manifest refraction (sphere and cylinder), and HOAs using propensity score matching. 

In our study, both TG-LASIK and SMILE showed no significant difference in postoperative visual acuity or SEQ. Importantly, our findings showed that SMILE induced significant increases in SA, coma, and residual HOAs (from the third-order up), whereas TG-LASIK did not. At the six-month follow-up, the TG-LASIK group showed statistically significantly lower coma and residual HOAs, except SA, than those of the SMILE group. HOAs play an important role in visual quality. Corneal aberrations account for nearly 80% of all aberrations of the eye and significantly influence visual quality [1,26]. In the Zernike HOA chart, relatively low order primary coma and relatively central axial position primary SAs have the most obvious influence on visual quality [27,28]. Therefore, our results suggest that the use of TG-LASIK promotes better visual quality than does SMILE.

Only a few studies have previously analyzed the induction of HOAs after LASIK and SMILE [3,4,23,25]. Gyldenkerne et al. investigated differences in corneal HOAs between SMILE and FS—LASIK; three months postoperatively they found that aberration changes in the total cornea were significantly higher in the FS-LASIK group compared with in the SMILE group [4]. Lin et al. compared 60 eyes treated with SMILE (mean SE: −5.13 ± 1.75 diopters) with 51 eyes treated with FS-LASIK (mean SE: −5.58 ± 2.41 diopters) and found significantly lower ocular total HOAs and spherical aberration in the SMILE group [25]. Liu et al. found that SMILE had a lower induction rate of spherical aberration in a 6 mm diameter analysis than that of FS-LASIK at six months postoperatively [23]. These studies reported that SMILE in general had significantly less SA induction, although there was no difference in coma, suggesting that it preserves corneal asphericity better than FS-LASIK [4,23,25]. These studies reported differed from the present study, which is thought to be due to differences in the LASIK ablation technique. Unlike conventional LASIK, customized ablation such as TG-LASIK or wavefront-guided LASIK (WF-LASIK) corrects not only manifest sphere and cylinder but also corneal irregular astigmatism and aberration.

Ye et al. reported that both SMILE and WF-LASIK showed advantages in inducing fewer total HOAs compared with LASIK and FS-LASIK [3]. In addition, similar to the present study, SMILE induced more horizontal vertical comas compared with WF-LASIK. This is because WF-LASIK also customized ablation. However, unlike the present study, WF-LASIK showed a significant induction of total HOA, SA, and horizontal coma compared with before surgery. The previous prospective contralateral-eye study using the latest laser platform, the same as ours, confirmed that TG-LASIK induced fewer HOAs and significantly decreased ocular trefoil, corneal total HOAs, and coma, although TG-LASIK and WF-LASIK provided similar refractive and visual outcomes [15]. This is because TG-LASIK differs from WF-LASIK in several ways. TG-LASIK is a topography-guided custom ablation treatment designed to produce an optimum corneal curvature by measuring the corneal topography. Measurement of the corneal curvature in TG-LASIK is not limited by the pupil, in contrast to wavefront measurements. Topography-guided custom ablation treatment is not susceptible to errors created by the pupil centroid shift as the pupil changes size, and it allows correction of peripheral corneal abnormalities on the cornea, where most HOAs of the optical system of the eye arise [29]. Aberrometer measurements are also affected by the accommodation and media opacities. WF-LASIK might not achieve optimal results because lenticular aberrations tend to change with the state of accommodation. Most recently, a meta-analysis by Hu et al. also demonstrated that both TG-LASIK and WF-LASIK were effective for myopia, but TG-LASIK produces fewer aberrations and better postoperative predictability than WF-LASIK [30].

In this study, we found that corneal coma decreased after TG-LASIK, although the difference was not statistically significant. Other studies using T-CAT LASIK reported equivalent findings [31]. This proves that the ablation was well-centered to the corneal vertex, and that the preoperative corneal HOAs were treated to some extent by TG-LASIK. If topography-guided LASIK was not performed, then the postoperative aberrations would have worsened because a symmetric ablation would have been performed without any regard for the preoperative irregular astigmatism or aberration. This also explains the significant increase in coma, SA, and residual HOAs after SMILE. SMILE is generally considered to engender few postoperative SAs. In fact, it seems the only aberration not showing a significant difference between the TG-LASIK and SMILE groups at the six-month follow-up was SA. However, all other aberrations were significantly decreased in the TG-LASIK group compared with the SMILE group. Therefore, TG-LASIK is considered advantageous for people with severe astigmatism or an irregular cornea. The preoperative astigmatism and the total RMS of the TG-LASIK group before PSM were significantly greater than those of the SMILE group. TG-LASIK was seemingly chosen by more people due to the advantages associated with accurate correction of the astigmatic axis via correction of a cyclotorsion and irregular cornea. This preoperative difference between the two groups was analyzed by PSM, thus minimizing selection bias that may be introduced when comparing the induction of aberrations between the two groups.

This study has some limitations. First, the sample size of the TG-LASIK group was small, and the patients were retrospectively selected. Second, a slightly larger optical zone was used in the TG-LASIK procedure than in the SMILE surgery. However, these differences did not have a significant impact on our results. Third, the difference in the quality of vision perceived by the patients was not assessed. It can be inferred that the quality of vision after TG-LASIK is better than that after SMILE surgery because there was no significant HOA induction after the surgery. However, the subjective improvement in the patients’ symptoms must be verified in order to state that absence of significant HOA induction is associated with an improved quality of vision. The refractive results may not necessarily match with the postoperative vision perceived and the overall patient satisfaction because non-refractive or non-visual factors are equally important. Fourth, only the corneal HOAs were measured, while the ocular HOAs were not. Both the corneal HOAs and the internal HOAs induced by the lens must be taken into consideration for the measurement of ocular HOAs. However, refractive surgeries mainly change the shape of the anterior corneal surface, not the lens shape. Therefore, the most marked change after surgery is noted in the corneal HOAs. Furthermore, compared with the measurement of ocular HOAs, the measurement of corneal HOAs is more advantageous as it is more reproducible and accurate; this may be because the corneal HOAs are less affected by tears, pupil size, pupillary center position, or angle kappa.

In conclusion, TG-LASIK is expected to provide better quality of vision after surgery because it did not yield a significant increase in postoperative HOAs, whereas significant increases in coma, SA, and residual HOAs were observed after SMILE surgery. In particular, the incidence of coma after TG-LASIK showed a decrease compared with that before surgery, so it may be expected to be effective in treating preoperative coma. However, a large-scale study on the improvement of the patients’ subjective symptoms after TG-LASIK in comparison with other refractive surgeries is required to generalize and confirm these results. Authors should discuss the results and how they can be interpreted from the perspective of previous studies and of the working hypotheses. The findings and their implications should be discussed in the broadest context possible. Future research directions may also be highlighted.

## Figures and Tables

**Table 1:** Preoperative characteristics of the two groups after propensity score matching.

	SMILE PSM-Group (*n* = 45)	TG-LASIK PSM-Group (*n* = 45)	*p*-Value
Sex, male, %	44.4	46.7	0.832
Age, years	24.9 ± 4.86	25.9 ± 5.34	0.307
Sphere, diopter	−3.80 ± 1.47	−3.23 ± 1.66	0.092
Cylinder, diopter	−1.44 ± 0.68	−1.73 ± 1.09	0.096
SEQ, diopter	−4.52 ± 1.47	−4.10 ± 1.62	0.178
CDVA, logMAR	0.00 ± 0.00	0.0011 ± 0.0075	0.323
IOP, mmHg	14.2 ± 2.77	14.8 ± 1.91	0.235
Pupil size, mm	6.79 ± 0.58	6.79 ± 0.65	0.955
CCT, μm	554 ± 25.8	545 ± 24.4	0.079
Flat K, diopter	42.4 ± 1.39	42.1 ± 1.59	0.523
Steep K, diopter	44.4 ± 1.39	44.3 ± 1.77	0.836
Optical zone, mm	6.43 ± 0.18	6.5	
Vertical coma, μm	0.0392 ± 0.206	0.0129 ± 0.294	0.655
Horizontal coma, μm	0.0401 ± 0.152	0.0087 ± 0.170	0.331
Spherical aberration, μm	0.197 ± 0.0970	0.204 ± 0.0756	0.644
Total HOAs, μm	0.476 ± 0.145	0.577 ± 0.477	0.165

The values of continuous variables are presented as mean ± standard deviation. (SMILE, small-incision lenticule extraction; TG-LASIK, topography-guided laser-assisted in situ keratomileusis; SEQ, spherical equivalent; PSM, propensity score matching; CDVA, corrected distant visual acuity; logMAR, logarithms of the minimum angles of resolution; IOP, intraocular pressure; CCT, central corneal thickness; K, keratometry; HOAs, higher-order aberrations).

**Table 2:** Comparison of postoperative refractive parameters between the two propensity-score- matched groups.

	SMILE PSM-Group (*n* = 45)	TG-LASIK PSM-Group (*n* = 45)	*p*-Value
Sphere, diopter	0.139 ± 0.383	0.100 ± 0.378	0.653
Cylinder, diopter	−0.472 ± 0.234	−0.439 ± 0.262	0.562
SEQ, diopter	−0.0974 ± 0.369	−0.119 ± 0.353	0.782
UDVA, logMAR	−0.0367 ± 0.121	−0.0722 ± 0.0627	0.087
Steep K, diopter	39.1 ± 1.56	39.4 ± 2.05	0.527
Flat K, diopter	40.1 ± 1.70	40.1 ± 2.24	0.929

The values of continuous variables are presented as mean ± standard deviation. (SMILE, small-incision lenticule extraction; TG-LASIK, topography-guided laser-assisted in situ keratomileusis; PSM, propensity score matching; SEQ, spherical equivalent; UDVA, uncorrected distant visual acuity; K, keratometry).

**Table 3:** Changes in corneal higher-order aberrations in both propensity-score-matched groups after each refractive surgery.

	Preoperative	Postoperative	Induced HOAs	*p*-Value
SMILE PSM-group				
Coma	0.239 ± 0.104	0.420 ± 0.216 ^a^	0.181 ± 0.233	<0.001
SA	0.197 ± 0.097	0.270 ± 0.133 ^b^	0.0736 ± 0.162	0.004
Trefoil	0.204 ± 0.134	0.251 ± 0.131 ^c^	0.047 ± 0.170	0.071
Residual HOAs	0.338 ± 0.132	0.489 ± 0.182 ^d^	0.151 ± 0.178	<0.001
TG-LASIK PSM-group				
Coma	0.305 ± 0.144	0.293 ± 0.214	−0.0118 ± 0.250	0.754
SA	0.204 ± 0.0756	0.214 ± 0.130	0.0100 ± 0.151	0.658
Trefoil	0.188 ± 0.091	0.196 ± 0.116	0.008 ± 0.154	0.721
Residual HOAs	0.396 ± 0.494	0.415 ± 0.117	0.0195 ± 0.506	0.797

The values of continuous variables are presented as mean ± standard deviation. (SMILE, small-incision lenticule extraction; TG-LASIK, topography-guided laser-assisted in situ keratomileusis; PSM, propensity score matching; SA, spherical aberration; HOAs, higher-order aberrations; ^a^ *p* < 0.05 vs. TG-LASIK PSM-group at postoperative 6 months; ^b^ *p* = 0.056 vs. TG-LASIK PSM-group at postoperative 6 months; ^c^ *p* = 0.057 vs. TG-LASIK PSM-group at postoperative 6 months; ^d^ *p* < 0.05 vs. TG-LASIK PSM-group at postoperative 6 months).

## Data Availability

The data of the present study are available on request from the corresponding author (Y.W.J.).
